# Urogenital tuberculosis in a patient with end-stage renal disease

**DOI:** 10.1590/0037-8682-0284-2019

**Published:** 2020-01-27

**Authors:** André Patrício Ferreira de Almeida, Dina Fabrício da Silva, Karla Cristina Silva Petruccelli, Juliana da Costa Matos, Rodrigo Xavier Moreira, Marcelo Cordeiro-Santos

**Affiliations:** 1 Fundação de Medicina Tropical Dr. Heitor Vieira Dourado, Manaus, AM, Brasil.; 2 Universidade do Estado do Amazonas, Manaus, AM, Brasil.; 3 Universidade Federal do Amazonas, Hospital Universitário Getúlio Vargas, Manaus, AM, Brasil.

**Keywords:** Tuberculosis, Chronic Kidney Disease, Urogenital

## Abstract

Tuberculosis is one of the most common infections worldwide with particularly high incidence rates in countries with unfavorable socioeconomic conditions and among persons with impaired immune systems. While most patients with this disease will present with pulmonary tuberculosis, immunocompromised individuals also commonly present with extrapulmonary manifestations. We report the case of a 28-year**-**old male patient with end-stage renal disease who presented with long-standing systemic symptoms and genitourinary manifestations, who was diagnosed with urogenital tuberculosis both by clinical and microbiologic criteria. Clinicians should always suspect tuberculosis in patients with chronic symptoms, especially in those with immunosuppression.

## INTRODUCTION

More than two billion people worldwide are estimated to be infected with tuberculosis (TB), with the highest incidence rates observed in countries with unfavorable socioeconomic conditions^1^. The risk of developing active TB is greater in persons suffering from comorbid conditions that impair the immune system such as end-stage renal disease^2^.

Although pulmonary tuberculosis represents the great majority of cases, extrapulmonary tuberculosis (EPT) is seen in about 10% of all cases. Urogenital involvement is the third most common form of EPT, just after lymph node and pleural involvement^3^.

There is an increased incidence of TB in patients with end-stage renal disease (ESRD) compared to the general population. In absolute numbers, this observation is especially important in areas where the disease is endemic as the presentation of TB in uremic patients is often unusual and insidious^4^.

Here we describe a patient with ESRD who developed urogenital and systemic symptoms, who was eventually diagnosed with EPT.

## CASE REPORT

The patient is a 28-year-old male student who is a known case of ESRD secondary to polycystic kidney disease and currently on maintenance hemodialysis who first presented to an Emergency Department in 2016 with a chief complaint of pain and swelling in the right testicle. Surgical drainage was performed which revealed abscess formation in the testicular pouch with preservation of the testis and epididymis. Intravenous antibiotics were administered after the completion of drainage. No information about material collection or culture results were available. 

**FIGURE 1:**
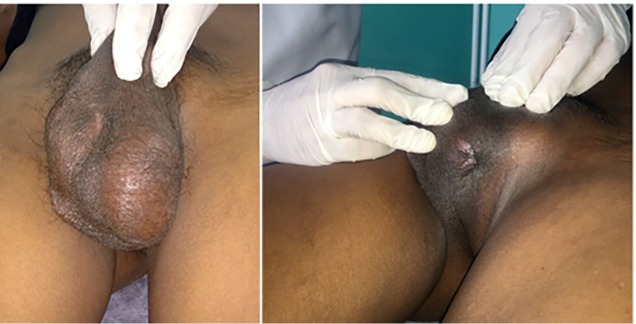
Left testicular swelling and fistulization with spontaneous drainage of small amounts of purulent fluid.

The patient then noted low-grade intermittent fever for almost two years, which was then followed by the development of an ulcerated lesion in the contralateral portion of the testicular pouch associated with purulent discharge (Figure 1). Other signs and symptoms noted at this time include diffuse mild abdominal pain, the appearance of a palpable mass in the mesogastric region and left hypochondrium, progressive difficulty in ejaculating, hematospermia, moderate pain and swelling in the left testicle, and occasional episodes of pyospermia. He was then referred to the ambulatory section of a tertiary hospital in Manaus, Brazil. 

Laboratory investigations showed negative results for HIV, hepatitis B, and hepatitis C. Urinalysis revealed pyuria and hematuria but without the presence of nitrites. Urine culture for common bacteria did not yield any growth. Serum measurement of the tumor markers beta-HCG, alpha-fetoprotein, and lactic dehydrogenase also yielded normal results.

**FIGURE 2:**
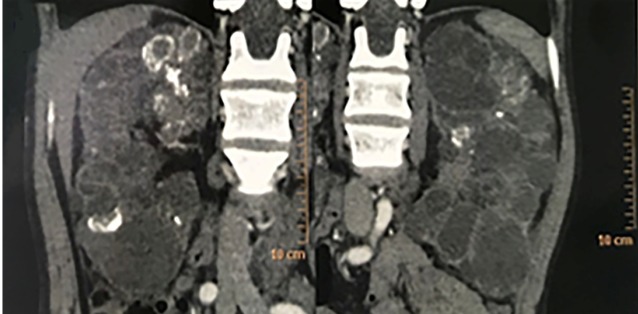
Coronal images of the patient’s abdominal CT scan showing enlarged kidneys secondary to the presence of numerous cysts.

A computed tomography scan of the chest and abdomen revealed minor pericardial effusion and small calcifications scattered in the liver, pancreas, spleen, and prostate. The kidneys had diffusely increased volume due to the presence of cysts disseminated in the parenchyma (Figure 2), the largest of which measured 4.7 cm in diameter. Lymphadenopathies were present in the retroperitoneum with some lymph nodes presenting with internal calcifications. 

On ultrasonography, the left testicle was found to be increased in size with a total volume of 32.5 cm^3^ containing numerous nodular and cystic structures with irregular borders and signs of hypervascularization; such findings suggest the presence of a chronic inflammatory or infectious process.

The tuberculin skin test was noted to be reactive, as demonstrated by a 12 mm induration after 48 hours, but acid-fast bacilli smear of the urine was negative. GeneXpert® MTB/RIF revealed the presence of Rifampicin-susceptible *Mycobacterium tuberculosis* (Mtb) while solid (LJ) and liquid (BACTEC MGIT 960 system) culture media showed growth of Mtb sensitive to first-line anti-TB drugs.

Anti-TB treatment using rifampicin (RIF), isoniazid (INH), pyrazinamide (PZA), and ethambutol (EMB) was then started with appropriate dose adjustments for creatinine clearance according to the Brazilian Guidelines for TB. The patient was monitored every month and a satisfactory outcome was eventually reached, as indicated by a reduction in the size of the abdominal lymph node mass and testicular edema, total cicatrization of the fistulous pathway in the scrotal sac, and the absence of fever starting from the first month of treatment.

However, during the third month of treatment, the patient developed painful nodules in the left epididymis and noted other symptoms which, when taken together, were compatible with retrograde ejaculation. Ultrasonography revealed a non-specific epididymal nodule while serum tumor markers for testicular neoplasms remained negative.

A multidisciplinary team approach was eventually used to manage the urogenital complications that arose secondary to TB cicatrization that developed in the patient. Full recovery was achieved after twelve months of TB treatment.

## DISCUSSION

The host response against intracellular pathogens, including *M. tuberculosis*, is determined by type 1 helper T-cell response and the production of interleukin (IL)-12, which increases the production of interferon (IFN)-ƴ. However, this mechanism is impaired in patients with chronic kidney disease^2^ ^,^ ^5^.

Proposed reasons for this decrease in cellular immunity include a defect in the costimulatory function of antigen-presenting cells, a persistent inflammatory state secondary to uremia and dialysis, vitamin D deficiency, hyperparathyroidism, and malnutrition^2^ ^,^ ^6^.

A negative tuberculin skin test, which occurs in 40-100% of cases of TB, can hamper the diagnosis of this disease^2^ ^,^ ^5^. However, the presence of a positive skin test in this patient aided the diagnostic process while bacteriologic and nucleic acid amplification tests (NAATs) results were still pending.

The classic finding of sterile pyuria is neither sensitive nor specific for tuberculosis. However, persistence of this finding should increase the clinician’s suspicion for TB^7^ ^,^ ^8^. NAAT is useful for both diagnosing and confirming tuberculosis in clinically suspected cases but the clinician must be cautious of false-negative results^2^ ^,^ ^8^ ^,^ ^9^. The patient presented with sterile pyuria but yielded positive results for NAAT and positive culture growth for TB late in the course of treatment.

There is no consensus regarding the treatment of TB in patients with renal impairment. However, some authors recommend using the standard six-month treatment regimen except in cases of meningeal, miliary or bone/joint tuberculosis. Others experts recommend a nine- or twelve-month-long regimen consisting of two months of treatment with INH, RIF, PZA, and EMB, followed by treatment with RIF and INH for the remaining months^2^ ^,^ ^3^ ^,^ ^5^ ^,^ ^7^ ^,^ ^9^.

Urogenital TB should be suspected in all patients presenting with chronic or insidious symptoms in any part of the genitourinary system, especially in those living in areas endemic for the disease.
